# The Effects of Nutrition on Chronic Conditions

**DOI:** 10.3390/nu15051066

**Published:** 2023-02-21

**Authors:** Omorogieva Ojo, Amanda Rodrigues Amorim Adegboye

**Affiliations:** 1 School of Health Sciences, University of Greenwich, Avery Hill Campus, London SE9 2UG, UK; 2 Centre for Agroecology, Water and Resilience, Coventry University, Coventry CV8 3LG, UK; 3 Centre for Healthcare Research, Coventry University, Coventry CV1 5FB, UK

The effects of nutrition on chronic conditions, such as diabetes, obesity, heart disease, and stroke, continue to generate interest among researchers. This is based on the fact that diet is a modifiable risk factor [1]. The composition of diet, including the proportions and types of macronutrients and micronutrients, is a major contributor to chronic diseases [1].

The beneficial effects of nutritional interventions on chronic conditions have been well documented, although differences remain among researchers concerning their overall impact [1]. Evaluations of the role of nutrition in chronic conditions draw on diet’s effects on body weight, body composition, glycemic and insulin excursions, and vascular remodelling. The effect of diet in modulating gut microbiota dysbiosis is also an evolving area of research [2].

This Special Issue, entitled “Nutrition in Chronic Conditions”, aims to examine the effect of nutrition in the development, care, and management of chronic conditions. This Special Issue includes 11 original studies conducted in high- and middle-income countries, 3 systematic reviews with meta-analysis, and 3 literature reviews. This editorial provides an overview of the key findings of the papers published in this Special Issue. These papers are broadly divided into seven topics: the effects of diet on (1) insulin and glucose metabolism; (2) gut health; (3) brain and cognitive impairment; (4) infections, chronic conditions, malnutrition, and all-cause mortality; (5) obesity and dietary variables in postmenopausal women; (6) non-alcoholic fatty liver disease in mice, specifically the consumption of coffee; and (7) chronic conditions and COVID-19 infection.

## 1. Insulin and Glucose Metabolism

The literature on the use of diets with a low glycemic index (GI) and a low glycemic load (GL) in the management of diabetes in adult populations is vast. However, little is known if GI and GL peaks are related to glycemic control, particularly in young and healthy populations. Using a representative national school-based sample of students (12–17 years old) without diabetes, da Rocha et al. [3] investigated the association between dietary indicators of the quality of carbohydrate intake and markers of glycemic control. The authors found the GI of diet was better at predicting insulinemia, regardless of weight status, compared to the GL [3]. The authors argued that guidance on food consumption based on carbohydrate quality should be provided to adolescents as a measure of glycemic control, as higher GIs are highly associated with the intake of refined carbohydrates. Encouraging healthy lifestyle habits combined with a diet with low GI and low GL can also help control obesity and reduce the risk of developing type 2 diabetes [3].

Apart from the impact of low GI diets on metabolism, postprandial insulin, glucose, and triglyceride responses have been investigated. Louca et al. [4] found that individuals with hypertension had higher postprandial insulinemic and lipemic responses to two standardized test meals compared to the normotensive controls after adjustments for sex, age, and BMI. This effect was partially mediated by visceral fat mass. No significant difference was observed for postprandial glucose. These findings corroborate existing literature on the key role of visceral fat in metabolic syndrome [4].

A drastic and prolonged reduction in carbohydrate intake leads to the exhaustion of glucose reserves in the body, shifting metabolism into ketogenesis and inducing hepatic oxidation of fatty acids. This process produces ketone bodies, an important alternative to glucose as the body’s source of energy. Dilliraj et al. [5] conducted a review to explore current evidence regarding ketone bodies in relation to nutrition, metabolic pathways, signalling functions, and effects on clinical conditions. Based on the studies reviewed, ketone bodies, which are formed under normal metabolism in the absence of glucose, play a key role in controlling oxidative stress and inflammation, resulting in improved mitochondrial function and growth, energy rescue, and adaptative epigenetic control. However, clinical trials are needed to validate the results obtained from in vitro and in vivo studies as well as from animal models [5].

## 2. Gut Health

Ojo et al. [6] conducted a systematic review and meta-analysis of randomised controlled trials to evaluate the effects of almonds on gut microbiota, glycometabolism, and inflammatory markers in patients with type 2 diabetes. This review was conducted against the backdrop of rising global prevalence of type 2 diabetes and the recognition that nutritional interventions, including the use of almonds, which are rich sources of dietary fibre, essential minerals, protein, and monounsaturated fatty acids, may be effective in managing symptoms of type 2 diabetes.

Ojo et al. [6] found that an almond-based diet was effective in promoting the growth of short-chain fatty acid-producing bacteria and lowering glycated haemoglobin and body mass index in patients with type 2 diabetes. The nutrient composition of almond, such as high fibre content and low glycemic index, may be involved in the biological mechanism of its effect. However, almonds did not appear to have a significant effect (*p* > 0.05) on fasting blood glucose, postprandial blood glucose, inflammatory parameters, glucagon-like peptide 1, and Homeostatic Model Assessment of Insulin Resistance [6].

In a separate systematic review, Ojo et al. [7] carried out a network meta-analysis of randomised controlled trials. This review aimed to evaluate the effect of prebiotics and oral antidiabetic agents on the gut microbiome in patients with type 2 diabetes. Prebiotics are substrates (non-viable) that are resistant to gastric acid and intestinal absorption and are used selectively by host microorganisms, which leads to benefits [7]. Prebiotics may promote eubiosis of the gut microbiome and ensure glucose homeostasis in patients with type 2 diabetes. The network meta-analysis found that prebiotics significantly reduced (*p* < 0.05) glycated haemoglobin, compared to the control, in patients with type 2 diabetes. However, prebiotics and oral antidiabetic agents did not have a significant effect (*p* > 0.05) on the gut microbiome, body mass index, fasting blood glucose, and postprandial blood glucose [7].

## 3. Brain and Cognitive Impairment

Metabolic syndrome (MS) is a prevalent condition worldwide and is characterised by a cluster of conditions, including central obesity, hyperglycemia, insulin resistance, hypertension, and dyslipidemia. Insulin resistance, believed to be a key underlying mechanism responsible for MS, affects multiple tissues and organs, including the central nervous system, leading to cognitive impairment and Alzheimer’s disease (AD). However, the inverse relationship between MS and cognitive impairment has not been fully explored. Rojas et al. [8] reviewed studies investigating a new hypothesis suggesting that cognitive impairment plays a role in the development of insulin resistance and, consequently, the appearance of MS. The authors concluded that a bidirectional relationship between MS and cognitive impairment seems to exist. However, large-scale longitudinal studies are still required to establish a causal relationship between these two factors [8].

In another study, Sochocka et al. [9] investigated the effect of *Ginkgo biloba* extract (Egb) as an alternative therapy on the mechanisms of innate immune response of peripheral blood leukocytes (PBLs) in patients with AD. The authors found that EGb has advantageous properties for health management in older adults and AD sufferers, especially in women with AD [9].

## 4. Infections, Chronic Conditions, Malnutrition, and All-Cause Mortality

*Helicobacter pylori (H. pylori)* infection is the most common cause of gastritis and other gastrointestinal disorders worldwide. Habbash et al. [10] investigated whether there is an association between dietary habits and *H. pylori* infections among 200 Bahraini adults. The authors found that among *H. pylori*-infected individuals, the consumption of coffee, green tea, and honey was significantly lower compared to non-infected individuals. They also found that vitamin D deficiency was a risk factor for *H. pylori* infection (OR = 1.1; 95% CI: 1.05, 1.18; *p* < 0.001). The authors suggested that coffee, green tea, and honey intake might be protective against *H. pylori* infection [10]. However, given the retrospective, cross-sectional study design, no causal relationship between dietary factors and *H. pylori* infection could be inferred.

Zupo et al. [11] conducted a systematic review and meta-analysis of the prevalence of zinc deficiency among patients suffering from inflammatory bowel disease (IBD). Zinc is essential for cell growth, tissue repair, and immune function. The authors included 17 studies and estimated an overall pooled prevalence of 50% (95% CI 0.48–0.52). However, the reviewed studies showed high heterogeneity, *I*^2^ = 96% [11]. These studies were further divided into two groups: Crohn’s disease (CD) (*n* = 9) and ulcerative colitis (UC) (*n* = 8). The prevalence of zinc deficiency was higher in patients with CD (54%) compared to those with UC (41%). The results point out that one in two patients with IBD has zinc deficiency, which can play a role in the severity of the disease. Therefore, clinicians should monitor zinc levels and other trace elements in patients with IBD.

Naber and Purohit [12] conducted a review to explore the role of diet in the management of chronic kidney disease (CKD). The authors focused on the Dietary Approaches to Stop Hypertension, the Mediterranean diet, and the whole-food, plant-based diet for their potential role in delaying CKD progression. They found strong evidence supporting the relevance of diets, which meet the daily nutritional requirement of patients, in the prevention and progression of CKD, particularly the whole-food, plant-based diet without the inclusion of animal products.

Malnutrition is prevalent among patients with chronic heart failure (CHF) due to the lack of appetite, unintentional weight loss, impaired intestinal function, catabolic metabolism, and other comorbidities. Schuetz et al. [13] investigated the cost-effectiveness of an individualised nutritional therapy in 645 hospitalised patients with CHF. The authors found that the overall incremental cost-effectiveness ratio for the individualised nutritional therapy vs. no nutritional therapy was 2625 Swiss Francs per life day gained. They concluded that the intervention increased life expectancy at an acceptable incremental cost-effectiveness ratio [13].

Malnutrition and loss of muscle mass are also prevalent among patients with cancer. In clinical assessments, handgrip strength (HGS) is used as a proxy of overall muscle strength. However, there are no population-specific values for HGS, particularly among oncology patients. Tribolet et al. [14] proposed sex-specific values for HGS stratified by age and tumour entity and tested their prognostic ability. The authors validated the prognostic value of HGS with respect to long-term mortality in hospitalised undernourished patients with cancer [14], which might aid clinical decisions.

Kwon et al. [15] examined the association between the intake of dietary fibres and CVD and all-cause mortality in the general population and among those with hypertension, diabetes, and dyslipidemia in a 10-year longitudinal study. After adjustments for confounders, the authors found that a higher intake of fibres reduced the risk of both all-cause mortality and CVD mortality [15].

## 5. Obesity and Dietary Variables in Postmenopausal Women

There are controversial results regarding the relationship between obesity and bone metabolism. López-Gómez et al. [16] investigated the differences in bone turnover among 250 postmenopausal women with and without obesity and compared their risk of fracture at five years of follow-up. The authors found that a bone formation marker (P1NP) was higher in women without obesity compared to women with obesity. However, postmenopausal women with obesity showed lower marker levels of bone formation, especially at younger ages. On the other hand, older women with obesity showed higher markers of bone resorption. This might be due to a decrease in vitamin D levels in women with obesity irrespective of age, which is associated with a high parathyroid hormone (PTH) level. However, no significant difference in the risk of fracture based on BMI was observed (OR = 0.90; 95% CI 0.30–2.72; *p* = 0.85). The authors concluded that the potential protective effect of obesity on bone mass and osteoporosis needs to be further investigated in other studies [16].

## 6. The Influence of Coffee Consumption on Non-Alcoholic Fatty Liver Disease in Mice

Di Mauro et al. [17] examined the effect of coffee consumption on non-alcoholic fatty liver disease in mice. In particular, this study aimed to establish if the intake of coffee might influence the expression of long non-coding ribonucleic acid (IncRNAs) in the liver. In this study, 24 four-week-old male mice were housed randomly in cages. Following one week of acclimation, the mice were randomly assigned to 1 of the 3 diets for 12 weeks, including a standard diet, a high-fat diet, and a high-fat diet plus decaffeinated coffee solution. This study found that decaffeinated coffee was effective in modulating the expression of IncRNAs, which are involved in the key pathways in the onset and progression of non-alcoholic fatty liver disease [17].

## 7. Chronic Conditions and COVID-19 Infection

In 2020, healthcare systems around the world were challenged by the COVID-19 pandemic. During the pandemic, it was observed that age and pre-existing health conditions (e.g., cancer, asthma, cancer, obesity, and diabetes) were risk factors for negative COVID-19 infection outcomes. Several micronutrient deficiencies were also associated with a higher risk for severe clinical symptoms. Voelkle et al. [18] found a heightened prevalence of micronutrient deficiencies (e.g., selenium, vitamin D, vitamin A, and zinc), particularly in older patients hospitalised for COVID-19. These deficiencies were also associated with more severe COVID-19 infection. The authors highlighted the need for further research regarding the effect of micronutrient supplementation on the treatment and prevention of COVID-19 infection [18].

The lockdown policies adopted by many countries to control the spread of the virus had a major impact on people’s lifestyles. Arayess et al. [19] observed that a personalised lifestyle intervention in children with overweight and obesity was less successful in decreasing BMI z-score during the COVID-pandemic compared to the same intervention one year prior to the first lockdown in the Netherlands [19].

## 8. Conclusions

Based on the above research findings, it is clear that nutrition plays an important role in the development and severity of chronic conditions in children, adults, and older adults. Therefore, healthy dietary patterns should be promoted, and further research should be conducted to fully understand the biological pathways regarding how diet may influence chronic diseases.
